# Exploring the Relationship between Mechanical Properties and Electrical Impedance in Cement-Based Composites Incorporating Gold Nanoparticles

**DOI:** 10.3390/ma17163972

**Published:** 2024-08-09

**Authors:** Daniel A. Triana-Camacho, David A. Miranda, Jorge H. Quintero-Orozco

**Affiliations:** Escuela de Física, Universidad Industrial de Santander, Cra 27 Calle 9, Bucaramanga 680002, Colombia; dalemir@uis.edu.co (D.A.M.); jhquinte@uis.edu.co (J.H.Q.-O.)

**Keywords:** cement paste, electrical impedance spectroscopy, electrical model, gold nanoparticles, mechanical properties

## Abstract

Structural health monitoring applications have gained significant attention in recent research, particularly in the study of the mechanical–electrical properties of materials such as cement-based composites. While most researchers have focused on the piezoresistive properties of cement-based composites under compressive stress, exploring the electrical impedance of such materials can provide valuable insights into the relationship between their mechanical and electrical characteristics. In this study, we investigated the connection between the mechanical properties and electrical impedance of cement-based composites modified with Au nanoparticles. Cylindrical samples with dimensions of 3 cm in diameter and 6 cm in length were prepared with a ratio of w/c = 0.47. The Au nanoparticles (Au NPs) were synthesized using pulsed laser ablation in liquids, and their size distribution was analyzed through dynamical light scattering. Mechanical properties were evaluated by analyzing the Young modulus derived from strain–stress curves obtained at various force rates. Electrical properties were measured by means of electrical impedance spectroscopy. The experimental results revealed a notable reduction of 91% in the mechanical properties of Au NPs-cement compounds, while their electrical properties demonstrated a significant improvement of 65%. Interestingly, the decrease in mechanical properties resulting from the inclusion of gold nanoparticles in cementitious materials was found to be comparable to that resulting from variations in the water/cement ratios or the hydration reaction.

## 1. Introduction

Cement is a crucial material for the construction industry, exhibiting remarkable performance and durability under various environmental conditions [1,2]. To further enhance its mechanical, thermal, and electrical properties, it is imperative to explore the intricate structure of cementitious materials [3,4]. Improving the electrical properties of cement-based composites could delve into different practical features for structural health monitoring (SHM) applications, such as piezoresistive sensing characterized by direct current measurements, and cyclic compressive or tensile experiments [5,6]. The piezoresistivity of cement-based composites is attributed to the formation of new conductive paths produced by the conductive inclusions when a mechanical strain is conducted. Consequently, the electrical resistance decreases after a compressive strain and increases under tension. In addition, researchers have found correlations between the hydration process in cementitious materials and electrical impedance measurements [7], becoming a useful tool to follow the microstructure evolution in cementitious materials. Conversely, the thermal changes in conductive cement-based composites produced by external low voltages [8] can be useful in cold regions, reducing gas consumption. The fundamental composition of cement pastes includes calcium hydroxide (portlandite), aluminates, and non-hydrate cement (clinker) embedded within a nanostructured amorphous hydration product known as calcium silicate hydrates (C-S-H) (CaO)*_x_*(SiO_2_)(H_2_O)*_y_*. Even after the curing stage, cement paste is considered a heterogeneous structure, which, depending on its composition, preparation, and treatment, starts its service life with varied mechanical properties. Additionally, significant research focused on incorporating novel materials of micro or nano sizes has appeared in the last two decades [9]. Thanks to a good affinity between the ionic ligands in cement-based products with these micro and nanofillers, it has been possible to strengthen and optimize the cement structure [10], even infusing electrical properties that researchers are interested in incorporating in real structures of civil engineering for damage detection or strain-sensing applications [11]. Consequently, these improvements increase the complexity of modeling, therefore understanding the interactions that take place within cement-based composites [12].

Diving into the properties conferred by these fillers, zeolites and metallic oxides as nano-silica have shown notable benefits for cementitious materials, for example, reducing water content and shorter curing times in concretes [13]. These processes have been predicted by physicochemical approaches explored through the hydrophobicity of cement-based composites [14]. As a result, these oxides have impacted the hydration process. Finally, the addition of nano-TiO_2_ concentrated at 5 wt.% can accelerate the hydration process equivalent to the normal drying shrinkage experienced by a sample over one month [15]. It is important to note that no metallic particles were detected in these studies or models. On the other hand, the multi-walled carbon nanotubes (MWCNTs) have been introduced into cementitious materials to enhance their thermodynamic [16] and electrical properties, also covering physical models to explain such electrical interactions between MWCNTs, the cement matrix and pores [17]. Furthermore, the studies conducted by Echeverry et al. [18] and Mendoza-Reales et al. [19] have delved into the influence of dispersing agents to control the mechanical properties of MWCNTs-cement composites. Regarding other carbonaceous nanocomposites, the influence of reduced graphene oxide (rGO) on the physicochemical properties of the cement matrix is also being modeled. The affinity between Ca^2+^ ions and carboxylate groups (COO^−^) present in rGO acts as a bridge, enhancing the mechanical strength of the cement matrix [20]. Moreover, this suggests that models are crucial for understanding the impact of these materials on cement-based composites capabilities.

It has been observed that mechanical and electrical properties have gained considerable significance with future purposes of structural health monitoring (SHM) applications. In that sense, carbonaceous fillers are commonly utilized to enhance the piezoresistive capabilities of cement-based composites [6], quantified by fractional change in resistance (FCR), which directly depends on strain instead of pressure variations when cyclic compressive stress is applied side by side in cubic specimens [21]. Furthermore, the piezoresistive effect can detect impacts caused by metallic spheres, while a cyclic loading is conducted [22]. Furthermore, the concentration of carbon fibers plays a critical role in determining the magnitude of changes in relative resistance and the gauge factor [23]. In comparison, Han et al. [24] observed an FCR up to 10.8% at a compressive stress of about 8 MPa, with CNTs concentration at 0.2% by cement volume. In addition, metal oxides and ceramics, such as lead zirconium titanate (PZT), are commonly used to improve the piezoelectric properties of cement composites [25]. These properties are commonly evaluated using the piezoelectric charge factor d_33_ and the voltage factor g_33_. Yet, the challenge has been incorporating new approaches or models to describe these parameters in the context of cement-based composites [4], with the aim of improving and standardizing the elaboration of these smart construction materials. For instance, the model presented by Triana-Camacho et al. [26] reveals inductive mechanisms in rGO-cement sensors and demonstrates that the d_33_ factor of this material (up to 1122 pm/V) could position it as a strong candidate for leading the development of piezoelectric-based cement sensors. These innovative electrical properties are closely interconnected with the mechanical properties of cement composites, making their evaluation imperative for practical applications. For that reason, several studies have observed that nanocomposites can enhance mechanical strength compared to reference specimens made of cement or concrete. For example, Ahmadi et al. [27] reported a notable improvement of 26% in compression resistance when incorporating zeolites at a concentration of 20% by weight and curing the samples for 28 days. On the contrary, Caputo et al. [28] published a decrease in compression resistance ranging from 12% to 45% when using zeolite concentrations of 5 wt.% to 25 wt.%. More recently, Imanian Ghazanlou et al. [29] conducted a complete study including the mechanical properties, electrical conductivity, thermal conductivity, and chemical properties of the rGO@Fe_3_O_4_cement composites. The main physicochemical findings showed how the hydration process of the hardened cement paste is affected. This mechanism had an incidence in the more compact rGO@Fe_3_O_4_-cement structure, presenting the maximum mechanical strength for the concentrations of rGO at 0.1 wt.% and rGO@Fe_3_O_4_ at 1.0 wt.%.

Most of the research on cement-based composites incorporating electrical impedance spectroscopy has focused on monitoring corrosion in cement. Later, with the inclusion of fibers in the cement, the focus shifted to studying the cement microstructure according to the incorporated dispersions [30]. These findings have paved the way for applications in SHM. For example, today, we know that the methodology for measuring electrical resistance depends on both the type of measurement, alternating current (AC) or direct current (DC), and the electrode configuration [31]. The main difference is that in DC mode, large voltages (on the order of V) are required to stimulate the material because it is necessary for the electric charge to move over long distances between the electrodes. In contrast, in AC mode, small voltages (on the order of mV) are sufficient to stimulate the material because the goal is to determine the material’s response to different frequencies of the applied voltage oscillation. For instance, the low energy consumption and sensitivity of the frequency response make it possible to detect the damage index in a structure through related indices [32].

Up until now, the applications of metallic nanoparticles have been mostly limited to producing anti-aging, antiseptic, purified air composite paint, or other ecological building materials [33]. Recent research indicates that incorporating nanoparticles such as nano-SiO_2_, nano-TiO_2_, and nano-Fe_2_O_3_ can significantly enhance the performance and durability of concrete. Nano-SiO_2_ particles, for example, contribute to the formation of additional C-S-H structures, while TiO_2_ nanoparticles, with their photocatalytic property, act as a self-cleaning agent in concrete. The inclusion of nanoparticles in concrete aims to serve as nuclei for new C-S-H phases, leading to increased microstructure density and reduced porosity [34]. Through a literature review, it has been determined that the use of gold nanoparticles (Au NPs) in cement-based composites is not widespread, as they are used primarily as biomarkers or biosensors, as exemplified by the work of Wang et al. [35]. However, in other materials, the incorporation of Au NPs has shown promise in enhancing certain piezoelectric properties. For example, Pusty et al. [36] successfully demonstrated the recovery of piezoelectric properties in polymers when Au NP was added, achieving maximum stress of 20 kPa and generating a voltage of approximately 6 V. Considering the focus of this study on understanding the electrical properties of the composite and the favorable characteristics imparted by the metallic nature of Au NPs, it is important to examine how each constituent material contributes to these properties. This involves evaluating the composite’s behavior under electrical impedance spectroscopy and assessing the mechanical resistance of Au NPs/cement-based composites to gain deeper insights into their overall electrical performance. Furthermore, nano-graphite, copper powder, and iron powder are indeed known for their conductive properties and lower cost. Au NPs offer unique advantages that justify their use in our research. (i) Although materials like graphite, copper, and iron are highly conductive, Au NPs can form an efficient conductive network within the cement matrix due to their nanoscale size and uniform distribution. This can lead to a more significant improvement in electrical conductivity compared to other materials [20]. (ii) Au NPs are chemically stable and resistant to oxidation, unlike copper or iron powders, which can rust over time. This stability enhances the long-term durability of the composite material, which is crucial for SHM applications [36]. (iii) Besides improving electrical conductivity, Au NPs can also positively influence other properties, such as piezoelectric capabilities, which are uncommon in materials like graphite or iron. This makes cement composites with Au NPs suitable for advanced sensor and actuator applications, such as pedestrian detection, SHM, and pavement deicing [37].

Therefore, this study aims to explore the effects of incorporating low concentrations of Au NPs on the electrical properties of cement-based composites through a physical model based on electrical measurements in AC. Simultaneously, this study considers the effects of such properties when the material is cured under a constant electric field. Currently, a big problem of piezoelectric cement-based composites is the loss of electrical power through the continuous loading cycles [38]. Therefore, we consider that the addition of metallic non-reacting nanoparticles can offer electrical carriers to augment the generating electrical current or even increase the capacitance for sensing purposes in SHM. On the other hand, copper slag has been used in cementitious materials, but its use has been limited to electromagnetic shielding applications [39,40]. There are no reports on its potential piezoresistive or piezoelectric properties in cement. Before exploring less commercial metallic materials such as copper, we wanted to use laboratory-grade reagents. Regarding the electric and mechanical properties, a series of compressive strength tests and electrical impedance experiments on cement composites based on gold nanoparticles were performed. Then, inverse modeling was employed through lumped electric circuits to gain deeper insights into the observed relationships between electrical and mechanical properties. Additionally, it has been implemented a non-conventional inverse modeling approach, wherein the impedance measurements were divided into three frequency domains and complex non-linear root-mean-square error (RMSE) minimization to extract the electrical parameters of the model, as described in the methodology section.

## 2. Materials and Methods

This section presents a description of the fabrication procedure for Au NPs in an aqueous solution, along with their size characterization, followed by the subsequent development of Au NPs/cement-based composites. Furthermore, the approach employed for mechanical characterization and electrical measurements in AC is outlined. The complete experimental setup to elaborate the Au NPs/cement-based composites has been previously published by Triana-Camacho et al. in the reference [41].

### 2.1. Materials

The physical properties of the materials used in this study are presented in Table 1.

**Figure 1:**
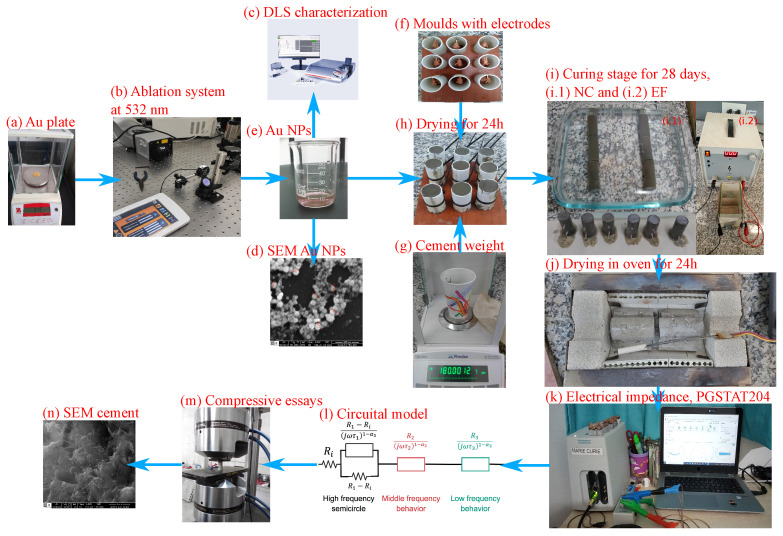
Illustrative scheme of the fabrication and characterization of cementitious compounds based on gold nanoparticles. The procedure begins with (**a**) weighing the gold plate. Next, in a beaker with 50 mL of water, the gold plate is deposited to initiate (**b**) the pulsed laser ablation process in liquid media (PLAL) as described in Table 2. Then, a small amount of the (**e**) solution with Au NPs is subjected to particle size analysis by (**c**) dynamic light scattering (DLS) and identification of the morphology of the Au NPs by (**d**) scanning electron microscopy (SEM) coupled to energy-dispersive spectroscopy (EDS). During the mixture preparation, 28.2 mL of Au NPs solution and (**g**) 60 g of cement were mixed to prepare each cement specimen. The mixture was poured into cylindrical molds with a height of 6 cm and a radius of 1.5 cm, which were poured inside (**f**) the molds. Additionally, the specimens were prepared according to the specifications required for compressive testing of cylindrical specimens [42]. These contained a pair of copper electrodes separated by 2 cm. (**h**) The samples were dried at room temperature for 24 h. After the initial drying, the specimens were subjected to (**i.1**) normal curing (NC), i.e., without an electric field (EF), and (**i.2**) curing under the influence of an EF for 28 days. The electric field was generated using two copper plane electrodes measuring 13 cm and 10 cm in length, respectively. Then, (**j**) the specimens were dried in an oven for 24 h at 50 °C until they were ready for (**k**) electrical characterization in AC. Based on the electrical impedance results, a script programmed in Python language was fed, which uses optimization algorithms such as least-squares or basin hopping, at the user’s choice. The optimization function is the electrical impedance of the (**l**) electrical circuit. Later, (**m**) compression loading tests on the cylindrical samples were performed at different scan rates. Once the specimens failed, (**n**) small pieces of 1 cm × 1 cm were subjected to morphological analysis by SEM.

The scheme in Figure 1 elucidates the process from the fabrication of Au NPs to passing through the manufacturing of gold nanoparticle-based cement specimens, characterization, and ending with the modeling and optimization of the electrical properties in AC. Furthermore, the size distribution and characterization of the Au NPs were then determined through appropriate techniques. Subsequently, the Au NPs were incorporated into the cement matrix to obtain Au NPs/cement-based composites, employing a well-defined sample preparation method developed for this study.

Following the preparation of the composites, a series of mechanical and electrical assays were conducted to evaluate their properties. Mechanical characterization involved performing specific tests to assess the response of the composites under various loading conditions. AC electrical measurements were carried out to analyze the electrical impedance behavior of the composites. To gain a deeper understanding of the observed electrical properties, a circuital model was used. This model, along with an algorithm, was utilized to extract the relevant parameters necessary for characterization. The algorithm employed a non-conventional approach, utilizing complex non-linear RMSE minimization. The detailed methodology for the circuital model and the extraction of its parameters are described in the subsequent sections.

### 2.2. Gold Nanoparticles

Au NPs were chosen over other metallic particles highly conductive, such as copper nanoparticles (Cu NPs), considering the next argument: (i) Gold is chemically inert and resistant to corrosion, making it more stable in the highly alkaline environment of cement. This stability is crucial for ensuring the long-term durability and consistency of the composite’s electrical properties. Copper, on the other hand, is prone to oxidation and corrosion, which can compromise the material’s integrity and electrical performance over time [43]; and (ii) Previous studies, such as those by Oumghar et al., have demonstrated that Au NPs can significantly enhance the piezoresistive and piezoelectric properties of polymer matrices. These properties are advantageous in cementitious materials, suggesting similar potential benefits in terms of mechanical and electrical performance [44].

The synthesis of Au NPs was achieved using the pulsed laser ablation in liquids (PLAL) method, which is known for its rapid nanoparticle production from solid materials. Various parameters that influence nanoparticle production were carefully controlled and are listed in Table 2. Two different ablation times, namely 5 min and 10 min, were used to obtain Au NPs with different particle sizes, which were dispersed in ultrapure water at concentrations of 442 ppm and 658 ppm, respectively.

**Figure 2:**
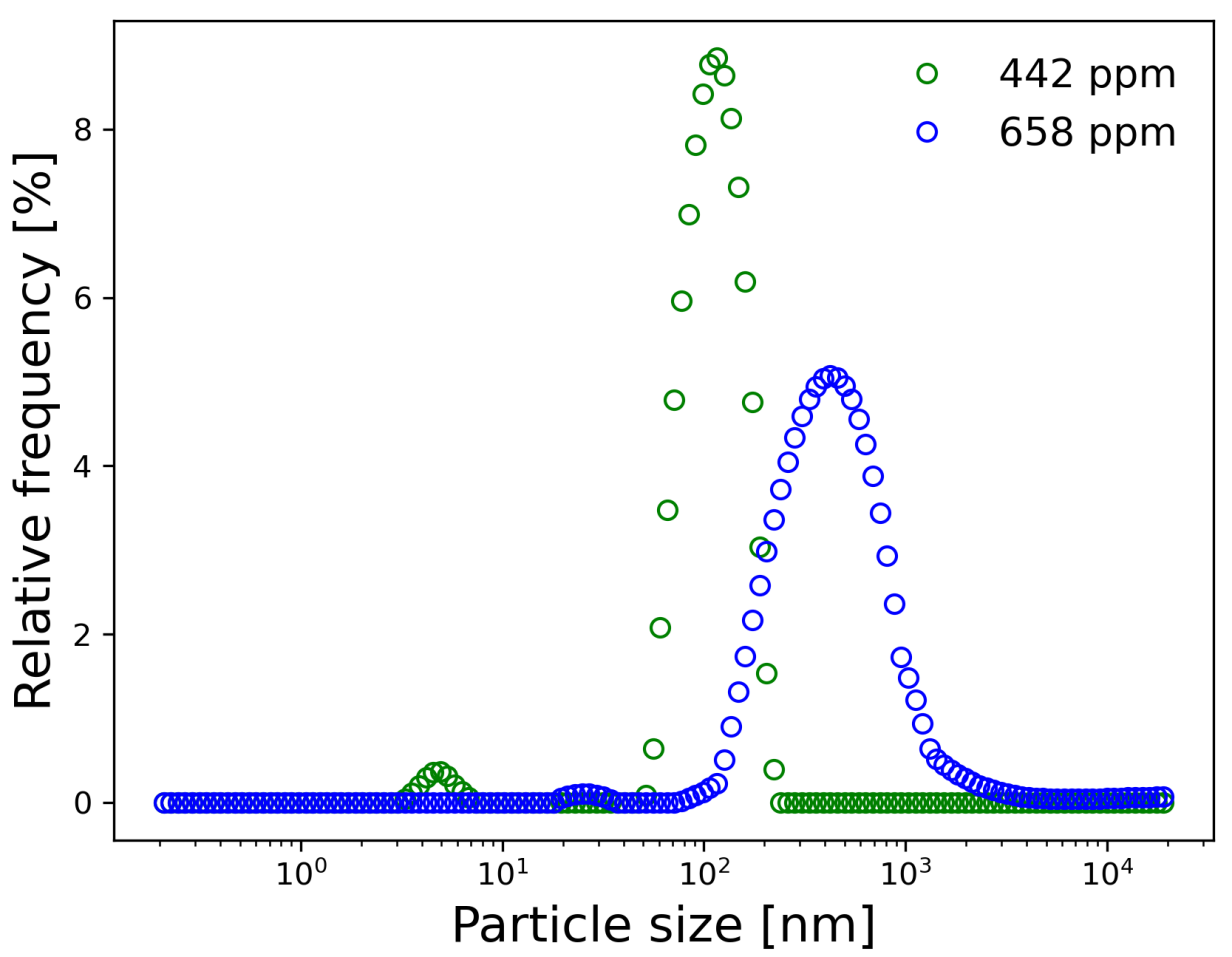
Particle size distribution of Au NPs dispersions with concentrations of 442 ppm and 658 ppm.

To determine the exact concentrations of the gold nanoparticles, the mass of the gold plate was weighed both before and after the PLAL process, with three replicates performed. Subsequently, the dispersion of Au NPs was diluted in a 1:100 ratio to conduct particle size distribution measurements using dynamic light scattering (DLS). The particle size distribution was determined in triplicate for each concentration at a scattering angle of 90° using a Litesizer 500 particle analyzer manufactured by Anton Paar. Figure 2 presents the resulting curves, which depict the relative frequency of Au NPs sizes for the specified concentrations. The curve corresponding to the 442 ppm concentration exhibited a prominent peak at 113 ± 4 nm, while the 658 ppm curve displayed a maximum at 425 ± 28 nm. These measurements indicate that shorter ablation times yield smaller Au NPs sizes. However, shorter ablation times also give rise to additional populations of Au NPs, observed around peaks at 1.62 ± 0.41 nm.

### 2.3. Gold Nanoparticles-Cement Composites

Ultrapure water obtained from the Milli-Q IQ 7000 purification system, with an electrical resistance of 18.2 MΩ, was used to prepare Au NP dispersions. Subsequently, Au NPs/cement-based composites were fabricated following the steps described below.

(i) The dispersions of Au NP and ordinary Portland cement were mixed with a water-to-cement ratio (w/c) of 0.47.
(ii) The resulting cement paste was then poured into cylindrical molds with dimensions of 6 cm in length and 3 cm in diameter.
(iii) The molds filled with cement mixed with Au NPs were subjected to 10 min of vibration using a vibration table.
(iv) Finally, the samples were dried for 48 h and subsequently cured for 28 days. The entire manufacturing process adhered to the guidelines specified in ASTM C349-18 [45], ensuring standardization.

Moreover, some of the samples underwent curing under the influence of an external electric field (EF) [41]. This was achieved by placing the samples between two parallel plates connected to a 20.1 V power source. Following the curing stage, the samples were further dried in a furnace at a temperature of 40 °C for 24 h. The nomenclature assigned to each sample is provided in Table 3.

### 2.4. Mechanical Performance

**Figure 3:**
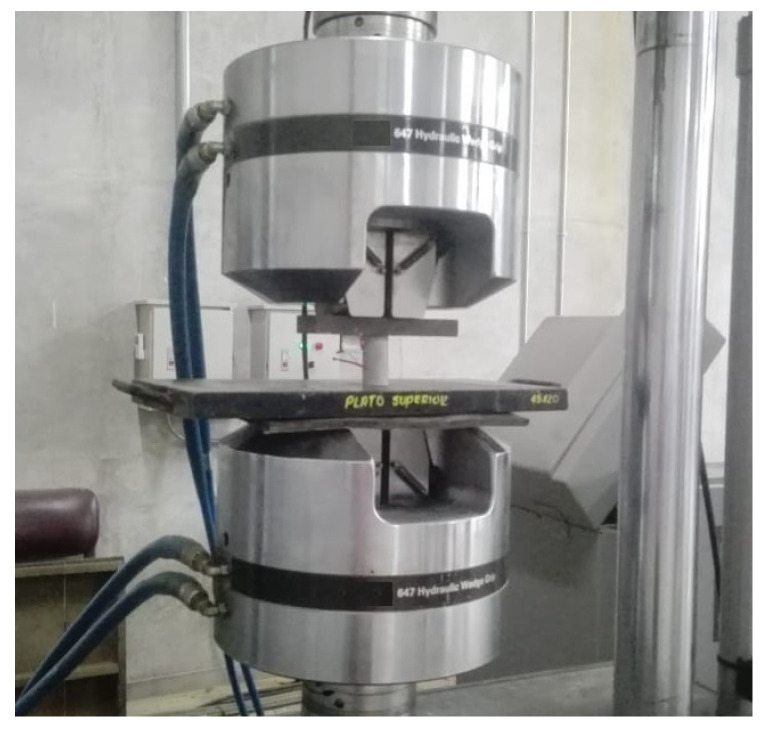
Compressive essay of Au NPs/cement-based composites.

Compressive strength tests were conducted using a universal testing machine model MTS-810 (with a maximum force capacity of 500 kN) until reaching a maximum deformation of 0.6 mm, as depicted in Figure 3. Initial tests on reference specimens were carried out at loading rates ranging from 0.1 kN/s to 0.3 kN/s while ensuring the material remained within its linear zone. This step aimed to identify the optimal loading rate for testing the Au NPs/cement-based composites. Subsequently, compressive strength tests were performed on the Au NP-cement composites at a loading rate of 0.2 kN/s until fracture occurred.

### 2.5. Electrical Characterization

The electrical properties in AC of the Au NPs/cement-based composites were evaluated using electrical impedance spectroscopy (EIS). Measurements were made with a voltage amplitude of 10 mV, and the frequency was swept from 0.1 to 1 MHz, with 60 evenly distributed points. The electrical data obtained were analyzed using Nyquist diagrams and Bode plots, where semicircles and dispersions were fitted using optimization algorithms to investigate the physics behind the electrical interactions between the Au NPs, cement background, and electrodes.

**Figure 4:**
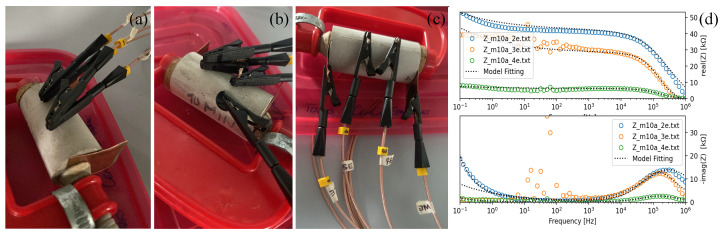
Electrical impedance measurements at two (**a**), three (**b**), four (**c**) electrodes, and (**d**) result of the calibration.

EIS measurements were conducted on specimens of Au NPs/cement-based composites using the calibration outlined in Figure 4. These measurements employed two-point (a), three-point (b), and four-point (c) configurations. The data were then compared to observe the noise, as depicted in the Bode diagram fitted to the model proposed in this study (see Figure 4d). In the Bode diagram, the blue curve represents the two-electrode measurement, the orange curve is the three-electrode measurement, and the green curve is the four-electrode measurement. The noise observed in the three-electrode measurement is attributed to the connection of the third electrode, which used conductive carbon tape, commonly utilized in scanning electron microscopy (SEM) or energy-dispersive X-ray spectroscopy (EDS) applications. Despite changes in impedance magnitude, the curve profile remains consistent, indicating that the sample’s physics is unaffected by the number of electrodes connected.

In electrochemistry, the reference electrode is placed near the working electrode to minimize electrolyte resistance, therefore enhancing measurement precision and reducing errors caused by concentration or potential gradients. As the reference electrode in the three-electrode configuration (orange in Figure 4d) is brought closer to the working electrode, the resulting curve approximates the two-electrode curve (blue in Figure 4d) due to increased electrode distance and impedance magnitude. The same effect is observed when increasing the distance between electrodes, which is fixed at 2 cm for all samples. Since the cement is hardened, there is no migration of species through the solution, and the resistance added by the electrode connections in two-point measurements reflects the compound’s physical characteristics. To calibrate the impedance spectroscopy measurements, three different methods were tested, including moving the electrode over the specimen. Determining the exact resistance of a cementitious compound is challenging unless a reference electrode like Ag-AgCl or the standard hydrogen electrode is used. Consequently, variations in electrical properties are expected across different samples used as references. The authors selected the least noisy measurements while preserving the specimens’ physical behavior.

**Figure 5:**
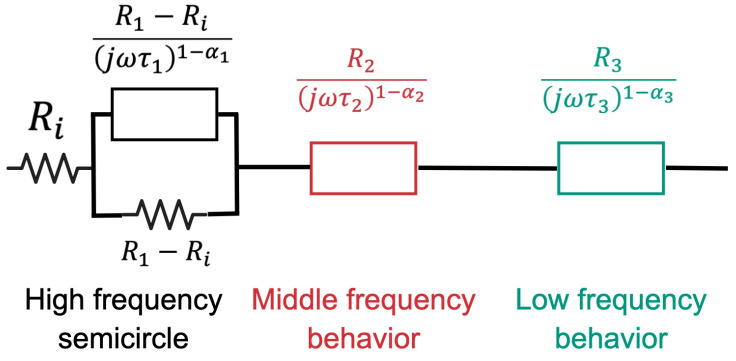
Circuital model used to fit the experimental data. In this model, three contributions are identified: the high-frequency contribution, which corresponds with a Cole–Cole semicircle model; the middle-frequency behavior, given by a CPE and the low-frequency behavior, given by another CPE.

The electrical impedance data were analyzed using a circuit model that accounted for three frequency ranges: low, middle, and high frequency (see Figure 5). Impedance measurements $Z_{k}$ were obtained at angular frequencies $\omega_{k}$ such that $\left(\omega_{k},Z_{k}\right)$, where k=1,2,⋯,n+1 and $\omega_{1}<\omega_{2}<\cdots <\omega_{n}$. Here, $\omega_{k}=2\pi f_{k}$, with $f_{k}$ denoting the frequency of sinusoidal excitation. The circuit model incorporated impedimetric Cole–Cole behavior at higher frequencies and constant phase elements (CPE) at low and middle-frequency ranges. The parameter extraction algorithm involved the following steps.

(i) Three cutoff frequencies $f_{\mathrm{low}}$, $f_{\mathrm{middle}}$, and $f_{\mathrm{high}}$ were selected such that $0<f_{\mathrm{low}}<f_{\mathrm{middle}}<f_{\mathrm{high}}<\infty$. The frequency ranges were defined as follows: $0\leq f_{k}\leq f_{\mathrm{low}}$ for the low-frequency range, $f_{\mathrm{low}}<f_{k}\leq f_{\mathrm{middle}}$ for the middle-frequency range and $f_{\mathrm{high}}\leq f_{k}<\infty$ for the high-frequency range.
(ii) Using the data in the high-frequency range, the parameters of the Cole–Cole equivalent circuit ($R_{1}$, $R_{i}$, $\tau_{1}$, and $\alpha_{1}$, as shown in Figure 5) were obtained by fitting the experimental data to a semicircle in the Nyquist plot. Parameter $\tau_{1}$ was optimized to minimize the fit error in the real and imaginary parts of the impedance.
(iii) Using all available data, the complex capacitance $C_{k}^{*}=1/\left(j\omega_{k}Z_{k}\right)$ was calculated at each frequency, and the Cole–Cole parameters $C_{\infty }$, ΔC, $\tau_{C}$, and $\alpha_{C}$ were obtained by fitting the experimental data to a semicircle in the Nyquist plot. The parameter $\tau_{C}$ was optimized to minimize the fit error in the real and imaginary parts of the capacitance. As a result of this step, the initial approximation of the low-frequency parameters in the circuital model was obtained: $R_{3}^{\left(1\right)}=\tau_{C}/\left(\Delta C+C_{\infty }\right)$, $\tau_{3}^{\left(1\right)}=\tau_{C}$, and $\alpha_{3}^{\left(1\right)}=\alpha_{C}$.
(iv) Using the high-frequency parameters ($R_{1}$, $R_{i}$, $\tau_{1}$, and $\alpha_{1}$), and $R_{3}^{\left(1\right)}$, $\tau_{3}^{\left(1\right)}$, and $\alpha_{3}^{\left(1\right)}$ as the initial approximation, a Levenberg–Marquardt optimization [46] based on the RMSE between the electrical impedance of the circuit model and the experimental data were performed. This optimization yielded the second approximation for the low-frequency impedance parameters, namely $R_{3}$, $\tau_{3}^{\left(2\right)}$, and $\alpha_{3}$. In this step, $R_{3}$ and $\alpha_{3}$ were fixed, while $\tau_{3}^{\left(2\right)}$ was re-optimized.
(v) Using the data from the middle-frequency range, a linear regression was applied to the Nyquist plot to obtain the slope *m*. The initial approximation for the parameters of the middle-frequency range was obtained as follows: $R_{2}^{\left(1\right)}=\max\left\{\mathrm{real}\left(Z_{k}\right)\right\}$, $\tau_{2}^{\left(1\right)}=1$, and $\alpha_{2}^{\left(1\right)}=1-2\pi^{-1}\tan^{-1}\left(1/m\right)$.
(vi) With the high and low-frequency parameters ($R_{1}$, $R_{\infty }$, $\tau_{1}$, and $\alpha_{1}$; $R_{3}^{\left(1\right)}$, $\tau_{3}^{\left(1\right)}$, $\alpha_{3}^{\left(1\right)}$; $R_{3}$, $\tau_{3}^{\left(2\right)}$, and $\alpha_{3}$) fixed and using $R_{2}^{\left(1\right)}$, $\tau_{2}^{\left(1\right)}$, and $\alpha_{2}^{\left(1\right)}$ as the initial approximation, a Levenberg–Marquardt optimization was performed to minimize the RMSE between the electrical impedance of the circuit model and the experimental data. This optimization provided the second approximation for the impedance of the middle-frequency range, i.e., $R_{2}$, $\tau_{2}^{\left(2\right)}$, and $\alpha_{2}$, where $R_{2}$ and $\alpha_{2}$ were fixed, and $\tau_{2}^{\left(2\right)}$ was re-optimized.
(vii) Using all available data and assuming fixed parameters except $\tau_{2}^{\left(2\right)}$ and $\tau_{3}^{\left(2\right)}$, a Levenberg—Marquardt optimization [47,48] of the RMSE was performed between the electrical impedance of the circuit model and the experimental data. This final step determined the values of $\tau_{2}$ and $\tau_{3}$ that minimized RMSE. In this step, $\tau_{2}^{\left(2\right)}$ and $\tau_{3}^{\left(2\right)}$ were used as the initial approximation for the Levenberg–Marquardt optimization.

The proposed model allows splitting the high, medium, and low-frequency contributions to electrical impedance. Furthermore, by breaking down the problem into parts, the relationship between parameters and the physics of electrical properties among cement-nanoparticles-electrodes was simplified.

## 3. Results

**Figure 6:**
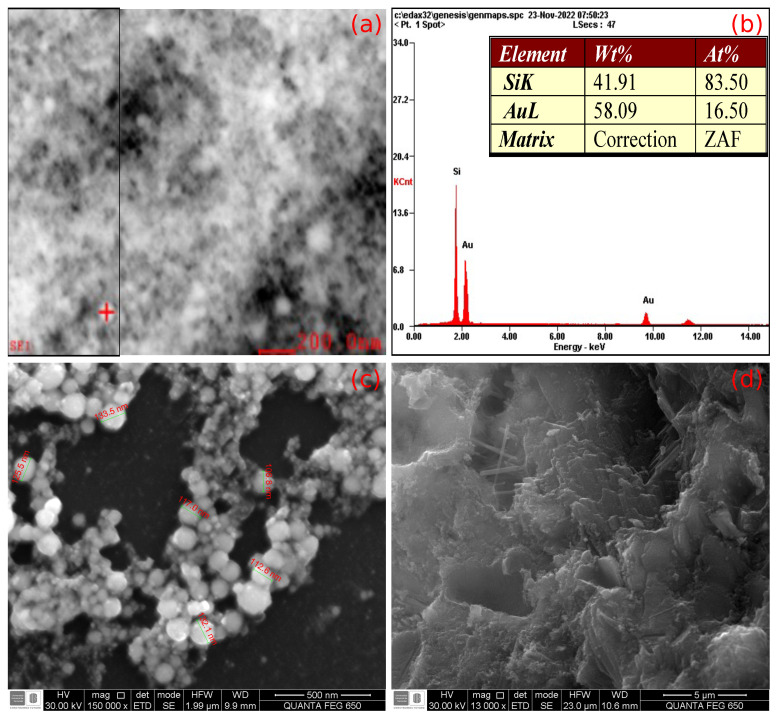
(**a**) Area where EDS was conducted, (**b**) EDS measurements to elucidate the presence of AU NPs in cement-based composites, (**c**) morphological characterization of Au NPs, and (**d**) cement-based composites using SEM.

The physical properties of cement powder largely determine the workability of the cement mixture. Additionally, small variations in the w/c ratio, particle size of aggregates, drying, and setting times also affect the final strength of the building material [49]. In this research area (cement-based composites), it has added aggregates of micrometric and nanometric sizes such as CNTs, graphene nanoplates, polymers, oxides, and metals [25]. In particular, carbonaceous materials functionalized with carboxylates have shown a strong affinity with calcium ions, which accelerates the formation of hydrates, impacting the final hardness of the cement [20]. In this case, gold is a noble metal, and it does not react with cement hydrates [50] because it is not solvated by water molecules as calcium silicates [51]. Instead, it should be accommodated in the pores or interstices of the cementitious material forming clusters such as Au NPs-loaded bone cement composites [52]. In Figure 6a,b, the EDS measurements confirm the Au NPs’ existence in the cementitious material. In contrast, it is possible to notice the spherical morphology and nanometric size of the Au NPs in Figure 6c and how they form conductive chains. Finally, a proper morphology of cement-based composite without excess pores families of huge sizes, which demonstrate a normal development of cement hydrates in the structure, is presented in Figure 6d. In that sense, the technical aspects of preparation and setting time (3, 7, and 28 days) provided by the manufacturer (ARGOS, Medellin, Colombia) were considered according to test standard NTC-220 [53]. Thus, a drying time of 24 h and a setting time of 28 days were defined, assuming that the inclusion of gold nanoparticles would not modify the workability and that cement would keep going with its properties as provided by the manufacturer on technical documentation. Also, previous experience with similar works using the same batch of cement with reactive inclusions, such as multi-layer carbon nanotubes [17] and reduced graphene oxide [26] was taken into account to navigate an avoidable study of the setting time for Au NPs/cement-based composites.

Fifteen cylindrical cement-based composites with Au NPs inclusions were prepared using two different methods: five samples were prepared using an external EF, and ten samples were prepared without an EF (see Table 3). The prepared samples were mechanically and electrically characterized, as described in the Materials and Methods section.

### 3.1. Mechanical Properties of Au NPs/Cement-Based Composites

**Figure 7:**
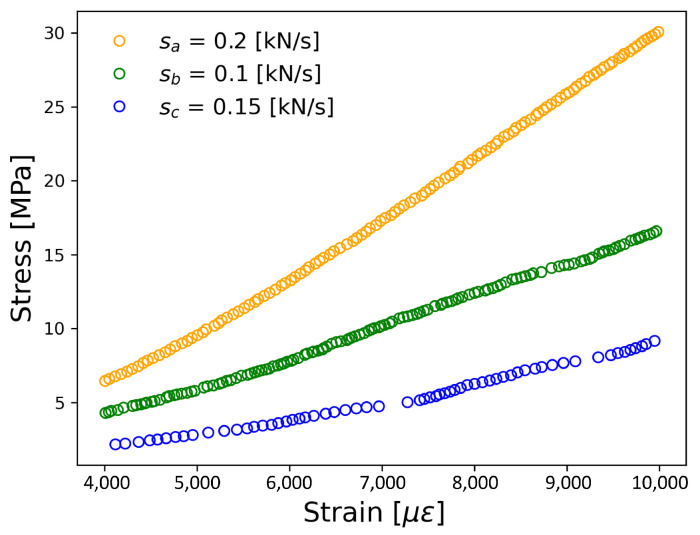
Stress-strain curves measured from reference samples to different load speed rates in kN/s. The Young’s modulus obtained to the different speed rates were $E_{a}$ = 4.06 GPa for $s_{a}$, $E_{b}$ = 2.11 GPa for $s_{b}$, $E_{c}$ = 1.21 GPa for $s_{c}$.

The stress-strain curves in the linear zone for different speed rates are presented in Figure 7. The maximum compressive strength ranges were 9 MPa to 30 MPa for cement samples, tested at speed rates of 0.25 kN/s and 0.2 kN/s, respectively. In comparison, reports in the literature indicate that cement specimens without the inclusion of nanomaterials exhibit compressive strengths of up to 48 MPa, with enhancements of up to 15% achieved with 0.05 by weight of the inclusions of CNT [54]. The other speed rates resulted in compressive strengths close to 16 MPa. Among the speed rates tested, the 0.2 kN/s rate demonstrated the best linear behavior, with a regression coefficient of 0.999. Furthermore, this speed rate yielded the highest Young’s modulus (E = 4.063 GPa). Based on these findings, the speed rate of 0.2 kN/s was selected for the compression tests on the Au NP/cement compounds.

**Figure 8:**
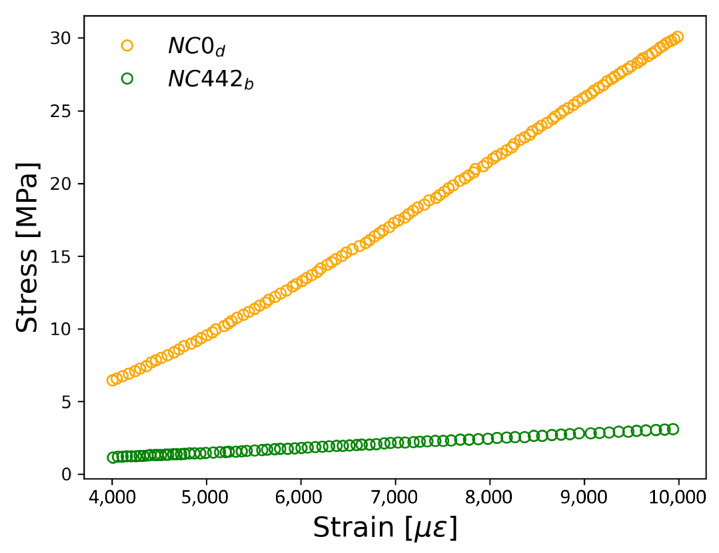
Stress-strain curves measured from reference (s4) and Au NPs/cement-based composites (s12). Both were measured at 0.2 kN/s.

When comparing the compressive strength curves of the reference samples and the Au NPs-cement composite samples (Figure 8), it is evident that the addition of Au NPs weakens the cement matrix since the maximum compressive stress decreases from 30 MPa to 2.2 MPa.

**Figure 9:**
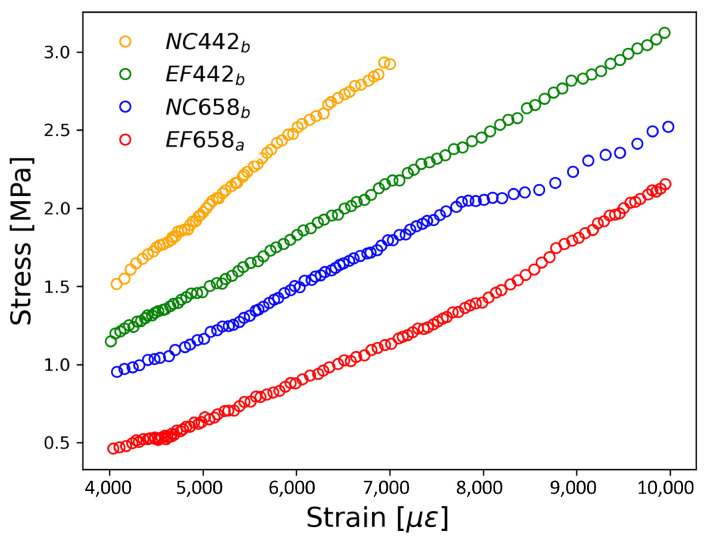
Stress-strain curves measured from Au NPs/cement-based composites. All were measured at 0.2 kN/s.

Figure 9 presents the stress-strain curves analyzed based on the concentration of Au NPs and the effect of curing under EF. When comparing samples based on the curing process, samples not cured under an EF (e.g., $NC442_{b}$) exhibit higher maximum compressive stress (3.1 MPa) compared to those cured under an EF (e.g., $EF442_{b}$) at a low concentration of Au NP (442 ppm). Samples with a high concentration of Au NPs (658 ppm) demonstrate the opposite behavior, where samples cured under EF achieve higher compressive stress than those cured in the usual way. For example, $EF658_{a}$ reaches 2.9 GPa at a strain of 7000 με, while the same sample without EF curing attains 1.2 GPa at the same strain. Furthermore, a comparison of samples based on the concentration of Au NPs reveals an opposite trend between samples cured under EF and those cured conventionally. Therefore, the high-concentration samples (at 658 ppm) cured under EF ($EF658_{a}$, $EF658_{b}$) exhibit the highest maximum compressive stress.

Young’s parameters were calculated from the stress-strain curves of all samples and are presented in Table 4. As expected, Young’s modulus of Au NPs/cement-based composites with lower concentration and cured under EF decreased by 91% compared to the reference samples. This decrement in mechanical properties can occur with the other nanofibers, for instance:

**Graphene Oxide (GO):** Similarly, the addition of GO can improve electrical conductivity but may reduce mechanical strength. Zhao et al. observed a decrease in compressive strength in cement composites with high concentrations of GO, which was attributed to poor dispersion and agglomeration of the nanoparticles [20].

**Nano-Silica:** The inclusion of nano-silica can enhance certain properties but also lead to a decrease in mechanical properties when used in higher amounts. Senff et al. found that high concentrations of nano-silica reduced the compressive strength of cement composites due to the increased porosity [55]. The reduction in mechanical properties can be attributed to the same mechanisms seen with other nanoparticles: disruption of the cement matrix, poor particle dispersion, and stress concentrations around the nanoparticles [21,24]. In addition, the addition of ${\mathrm{SiO}}_{2}$ nanoparticles to concrete samples has been found to cause a 14% reduction in compressive strength at high concentrations [56]. This decrease in mechanical properties is consistent with other studies that have examined the addition of other particles, such as nano-${\mathrm{TiO}}_{2}$, nano-clay, and CNTs, which also have shown a reduction in mechanical properties depending on the concentration of nanoparticles [25].

Regarding this research, the incorporation of Au NPs led to a significant decrease in both compressive strength and Young’s modulus. This reduction is consistent with the effects observed with other nano conductive fillers, indicating that the phenomenon is not unique to Au NPs but rather a general characteristic of nano-sized conductive additives [15,20,50].

The absence of carboxylate groups or other ionic chains in Au NPs limits their strong binding to the cement structure [20]. This can explain the significant reduction in Young’s modulus observed in Au NPs/cement-based composites, where the modulus decreased from 2.8 GPa for the reference samples to 0.27 GPa for samples with higher concentrations. Furthermore, the particle size of Au NPs can also have a positive or negative effect on the strength of the cement matrix [57]. The variations in particle size distribution observed in Figure 2 can be attributed to slight variations in the parameters of the pulsed laser ablation in liquids (PLAL) process used to synthesize Au NPs.

### 3.2. Electrical Properties of Au NPs/Cement-Based Composites in AC

**Figure 10:**
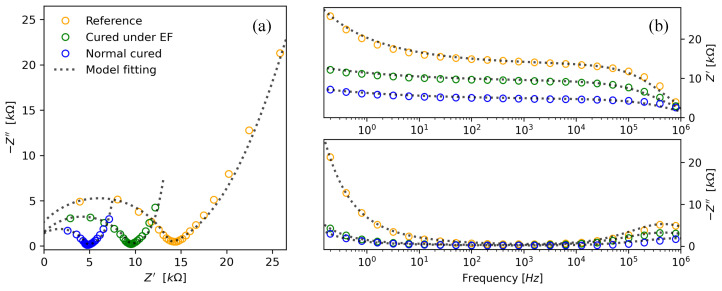
Electrical impedance spectra from reference and Au NPs/cement-based composites with a concentration of 442 ppm, where (**a**) is the Nyquist plots and (**b**) the Bode plots with $Z^{\prime }$ be the real part of the impedance and $Z^{\prime \prime }$ the imaginary part.

Regarding the electrical properties in AC, as shown in Figure 10, significant changes in high-frequency polarization resistance ($R_{1}-R_{i}$) can be observed when comparing the reference ($Ref_{5}$) and Au NP-doped ($NC442_{3}$) samples, Table 5. In this regard, the reference samples exhibit a high-frequency polarization resistance of approximately 15.3 kΩ, while the NC samples and those cured with an EF show values close to 5.9 kΩ and 10.3 kΩ, respectively. It is noteworthy that the high-frequency polarization resistance of the samples cured under an EF is almost twice that of the NC samples, despite the intention of enhancing their piezoelectric or piezoresistive properties through the application of an EF.

Zhou found that the percolation of cement composites with carbon nanofibers (CNF) is governed by the polarization phenomenon of CNFs, impacting electrical resistivity and temperature coefficient behavior. This study suggests that the polarization of CNTs significantly influences the electrical properties of cement composites [58] as well as, in this study has found out the induced polarization by an external electric field affects the transfer charge resistance as elucidated in Figure 10a. Meanwhile, Kim et al. found that adding carbon fibers improved the heat generation capability and electrical stability of cementitious composites with CNTs during heating, suggesting enhanced capacitive behavior at low and middle frequencies. This was possibly attributed to the formation of an overlapped area by adding the CF to the cementitious composites with CNT, which reduced the damage to the electrically conductive pathways induced by thermal expansion, additional hydration reactions, and internal cracking during the heating process [59], which could be corroborated through the here proposed model, for describing the electrical impedance.

Other parameters estimated from the circuit model described in Figure 5 are presented in Table 5. Specifically, the relaxation time $\tau_{3}$ and the heterogeneity factor $\alpha_{3}$ at low frequency (LF), the resistance $R_{2}$ at middle frequency (MF), and the relaxation time $\tau_{1}$ and the heterogeneity factor $\alpha_{1}$ at high frequency (HF) are highlighted.

The heterogeneity factor $\alpha_{3}$ tends to zero for samples containing gold nanoparticles. When approximating $\alpha_{3}$ to zero, the low-frequency CPE impedance $R_{3}/{\left(j\omega \tau_{3}\right)}^{1-\alpha_{3}}$ becomes approximately $R_{3}{\left(j\omega \tau_{3}\right)}^{-1}={\left(j\omega C_{3}\right)}^{-1}$. This indicates that the CPE at low frequencies exhibits a nearly pure capacitive behavior, which is given by $C_{3}=\tau_{3}/R_{3}$. The value of $C_{3}$ varies from 173 μF for the reference sample ($Ref_{5}$) to higher values of 881 μF for $EF442_{3}$ and 1306 μF for $NC44_{3}$. This capacitive behavior indicates an accumulation of electrical charges on both sides of the capacitors. The low frequencies correspond to near DC behavior.

At middle frequencies, the heterogeneity parameter $\alpha_{2}$ is close to 0.7, and a noncapacitive behavior is observed in the three electrically characterized samples. At these frequencies, the product of angular frequencies and the time constant is close to one, i.e., $\omega \tau_{2}\approx 1$. Since $\alpha_{2}$ is also close to one, we can interpret the behavior at middle frequencies as resistive and can be characterized by resistance $R_{2}$. Maurya et al. found that the presence of nanoparticles such as GO and CNTs can improve the integrity of the cement matrix by reducing water absorption and increasing resistance to acid and sulfate attacks [60]. This improvement in matrix integrity can be correlated with the polarization behavior observed at high frequencies, where the rapid electrical response is incompatible with ion movement and is more related to the internal structure of the cement matrix. The accumulation of charge in the copper electrodes and Au NPs can be compared to the resistance to chemical attacks observed in cement-based composites with GO and CNTs, where the ability of these nanoparticles to form a stable conductive network can contribute to charge accumulation and capacitive behavior at low frequencies.

**Figure 11:**
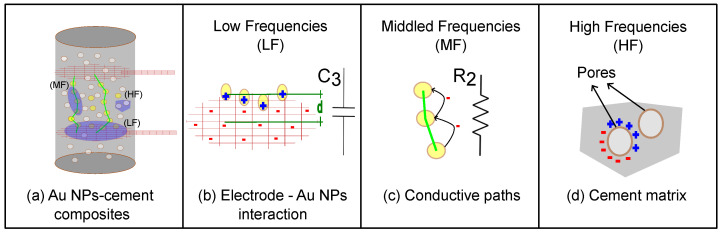
Model for describing the electrical impedance spectra of (**a**) Au NPs/cement-based composites and their physical mechanisms at (**b**) low, (**c**) middle, and (**d**) high frequencies.

In summary, a polarization behavior is observed at higher frequencies (HF), while a capacitive behavior dominates at low frequencies (LF). At middle frequencies (MF), a nearly resistive behavior is observed. As HF implies a rapid electrical response that is incompatible with ion movements, we could hypothesize that it is associated with the presence of pores in the cement matrix. The resistive behavior in MF could be associated with electrical conduction within the cement matrix, whereas the capacitive behavior in LF could be associated with the accumulation of charge in the copper electrodes and gold nanoparticles. This differentiation in frequency behavior can be schematically represented in Figure 11.

Additional commentary can be added to the schematic representation of electrical properties presented in Figure 11. In that sense, the electrical current flows between the two copper plates; these plates are represented by a red mesh, as shown in Figure 11a. Next, at low frequencies (LF), electrical interfaces play a significant role [61], and we speculate that the associated capacitance $C_{3}$ is due to the accumulation of charges near the electrode, as shown in Figure 11b. It is important to note that low-frequency capacitance can have two contributions in series: a geometric capacitance $C_{e}$ and a quantum (electrochemical) contribution $C_{q}$ [62]. Since Au NPs exhibit quantum effects due to the confinement of charge carriers, their energy levels are quantified and contribute to quantum capacitance [63].

As the current flows through the cement matrix between the copper electrodes, a resistive behavior dominates at middle frequencies (MF), characterized by resistance $R_{2}$, as depicted in Figure 11c. At higher frequencies (HF), the heterogeneity of the mixture comes into play, with contributions from the presence of pores in the cement, as illustrated in Figure 11d.

**Figure 12:**
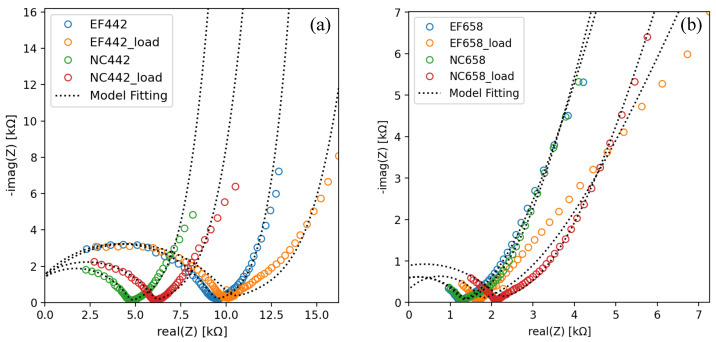
Nyquist diagrams of Au NPs/cement-based composites subjected under a compressive constant loading of 3 kN compared to non-compressed samples. Specimens are labeled as EF and NC according to their fabrication method and gold concentration at (**a**) 442 ppm, and (**b**) 658 ppm.

The effect on electrical impedance measurements when specimens doped with Au NPs were subjected to a constant compressive load of 3 kN will now be analyzed. Figure 12 illustrates how the compressive load modifies the shape of the Nyquist diagrams across three frequency ranges (low, medium, and high) for samples labeled as EF as well as NC. Additionally, it is observed that samples with a higher concentration (658 ppm) of Au NPs exhibit a lower impedance zone compared to those with a concentration of 442 ppm, confirming that there are more conductive paths in the samples with a higher concentration of Au NPs.

After fitting the model to the data in Figure 12, three parameters of those described above in Table 5 were selected. The selection criterion was that the parameter should exhibit the greatest sensitivity to the constant compressive force. Consequently, the chosen parameters were the charge transfer resistance $R_{1}-R_{i}$; the parameter $\alpha_{2}$, which was referred to above as the heterogeneity parameter because it defines the transition between pseudo-capacitive and pseudo-resistive behaviors of the CPE; and the relaxation time $\tau_{3}$, which contains information about capacitive behavior at low frequencies. Finally, these results are presented in Table 6. In every setup with EF or NC-labeled specimens, whether with low (442 ppm) or high (658 ppm) concentrations of Au NPs, applying compressive load increases the charge transfer resistance. Low-concentration samples (442 ppm) labeled NC increased this parameter by up to 33%, compared to 7% for those labeled EF. On the other hand, high-concentration samples (658 ppm) labeled EF increased by 65%. Similarly, those samples labeled NC changed by 64%. Thus, the effect of EF on this parameter is only noticeable at low concentrations. Hence, it is recommended not to apply an EF, at least not in the same direction as the electric current, to achieve greater sensitivity in this parameter. The heterogeneity parameter exhibited higher variations in specimens with a concentration of 442 ppm of Au NPs compared to those with a higher concentration of 658 ppm. The EF samples showed a minimal increase of 7%, whereas the NC samples showed a more significant increase of 18%. Once again, the greatest sensitivity is observed in the untreated samples under EF conditions. In contrast, the low-concentration samples both exhibited a shift of up to 3%. This parameter showed a minimum of 0.61 (NC) for the low-concentration samples, indicating a trend towards 1 in all cases, which suggests a pseudo-resistive behavior in the mid-frequency range. At low frequencies, the behavior of the samples was capacitive, as shown in Figure 11. Therefore, an increase in the relaxation time $\tau_{3}$ depicts an increase in capacitance. Then, in all configurations, capacitance increases when the compressive load is applied. Notably, specimens labeled as EF exhibit greater sensitivity than NC, increasing by 57% for low concentrations and 44% for high concentrations of Au NPs. In conclusion, the relaxation time of the CPE at low frequencies is highly sensitive to changes caused by mechanical compressive stress. The drawback, however, is that measurements at these low frequencies can take longer compared to high-frequency measurements.

## 4. Conclusions

In this study, the relationship between the mechanical and electrical properties of cement-based composites modified with Au NPs was explored through the application of advanced optimization algorithms. The focus was on understanding how these modifications impact both mechanical and electrical characteristics, extending beyond previous research that mainly concentrated on piezoresistive properties under compressive stress. The following key findings emerged from the research:

- The study revealed a significant 91% reduction in the modulus of elasticity for Au NP/cement-based composites compared to reference specimens. This reduction is not solely attributable to the Au NPs, as variations in the water/cement ratio may also contribute. Therefore, achieving a balance between mechanical properties and electrical improvements requires careful control of Au NP particle size and concentration.
- An improvement of up to 65% in AC electrical properties was observed. The addition of Au NPs led to contrasting effects on mechanical versus electrical properties.
- The findings highlight the usefulness of electrical impedance analysis for assessing the physical properties of aggregates within cement-based composites, such as bulk material, gold nanoparticles, and pores. This was facilitated by optimizing parameters across various frequency segments.
- The relaxation time at low frequencies ($\tau_{3}$) showed the highest sensitivity (57%) in specimens with a low concentration (442 ppm) of Au NPs when subjected to an electric field. This result suggests potential for further optimization in cost/benefit ratios.

Finally, the study’s findings emphasize the need for continued research to explore different experimental combinations and identify further correlations between parameters and design factors in cement-based composites. Future research should include a more in-depth first-principles analysis to provide statistically representative data on particle and pore sizes, aiming to optimize mechanical strength while maintaining desirable electrical properties. The insights gained lay a solid foundation for advancing the design and application of cement-based composites with enhanced properties.

## Figures and Tables

**Table 1:** Physical properties of Portland cement and gold.

Material	Density (g/cm^3^)	Size or Thickness (μm)	Resistivity (Ω× cm)	Purity (%)
Cement ARGOS	3.11	∼45	-	
Gold plate	19.33	200	2.35 × 10^−6^	99.999

**Table 2:** Parameters involved in Au NPs production at room temperature.

Materials	Laser Setup
Ultrapure water	Wavelength: 532 nm
(50 mL)	Spot diameter: 12.6 mm^2^
	Energy: 350 mJ
Gold plate	Ablation times: 5, 10 min
(99.9999% purity)	Time between pulses: 0.1 s
	Pulse duration: 8 ns

**Table 3:** Describing the samples involved the utilization of two methods: curing under an EF and without, referred to as NC. Three concentrations of Au NPs were considered: zero (reference), 442 ppm, and 658 ppm. The nomenclature employed here integrates the methods used for sample preparation (NC or EF), the concentration of Au NPs (0, 442, and 658 ppm), and a designated letter for sample identification.

Concentration	NC	EF
Reference	$NC0_{a}$, $NC0_{b}$, $NC0_{c}$, $NC0_{d}$, $NC0_{e}$	-
442 ppm	$NC442_{a}$, $NC442_{b}$, $NC442_{c}$	$EF442_{a}$, $EF442_{b}$, $EF442_{c}$
658 ppm	$NC658_{a}$, $NC658_{b}$	$EF658_{a}$, $EF658_{b}$

**Table 4:** Young’s modulus (*E*) from reference and Au NPs/cement-based composites.

Au NPs	*E* (NC)	*E* (Cured under an EF)
**Concentration**	**[GPa]**	**[GPa]**
Reference	2.800 ± 0.893	-
442 ppm	0.326 ± 0.003	0.260 ± 0.026
658 ppm	0.270 ± 0.009	0.346 ± 0.147

**Table 5:** Circuit parameters obtained by fitting the experimental electrical impedance spectra of reference and Au NPs-cement compounds with a concentration of 442 ppm to the circuit model of Figure 5.

Sample	$R_{i}$ [kΩ]	$R_{1}-R_{i}$ [kΩ]	$\tau_{1}$ [μs]	$\alpha_{1}$	$R_{2}$ [kΩ]	$\tau_{2}$ [s]	$\alpha_{2}$	$R_{3}$ [kΩ]	$\tau_{3}$ [s]	$\alpha_{3}$
$Ref_{5}$	−1.7	15.3	0.34	0.235	29.4	36.5	0.676	30.0	5.2	0.067
$EF442_{3}$	−1.1	10.3	0.34	0.293	9.8	158.9	0.750	13.4	11.8	0.000
$NC442_{3}$	−1.3	5.9	0.18	0.288	6.9	131.8	0.753	8.5	11.1	0.000

**Table 6:** Parameters obtained by fitting EIS measurements on Au NPs/cement-based composites, both under compressive loading and without. The sub-index “load” indicates the specimen was tested under a compressive load of 3 kN.

Sample	$R_{1}-R_{i}$ [Ω]	$\alpha_{2}$	$\tau_{3}$ [s]
EF442	10,339.25	0.74	11.42
$EF442_{load}$	11,078.96	0.69	17.95
NC442	5945.93	0.72	12.03
$NC442_{load}$	7939.23	0.61	14.84
EF658	2101.20	0.68	4.48
$EF658_{load}$	3469.22	0.66	6.47
NC658	2174.58	0.70	4.30
$NC658_{load}$	3571.02	0.72	5.57

## Data Availability

The research data and software can be found on GitHub repository https://github.com/dantrica/Streamlit_cement_impedance (accessed on 9 June 2024).

## Abbreviations

The following abbreviations are used in this manuscript:

AC	Alternating current
Au NPs	Gold nanoparticles
CPE	Constant phase element
EF	Electric field
HF	High frequencies
LF	Low frequencies
MF	Middle frequencies
NC	Normal curing
PLAL	Pulsed laser ablation in liquids
RMSE	Root-mean-square error
